# Resting-state heart rate variability after stressful events as a measure of stress tolerance among elite performers

**DOI:** 10.3389/fphys.2022.1070285

**Published:** 2023-01-04

**Authors:** Toshiya Miyatsu, Briana M. Smith, Andrew P. Koutnik, Peter Pirolli, Timothy J. Broderick

**Affiliations:** ^1^ Healthspan Resilience & Performance, Florida Institute for Human and Machine Cognition, Pensacola, FL, United States; ^2^ Department of Bioengineering, University of Washington, Seattle, WA, United States

**Keywords:** heart rate variabiity (HRV), assessment, stress tolerance, individual diffenrences, elite performance

## Abstract

**Introduction:** A common trait of elite performers is their ability to perform well when stressed by strong emotions such as fear. Developing objective measures of stress response that reliably predict performance under stress could have far-reaching implications in selection and training of elite individuals and teams. Prior data suggests that (i) Heart rate and heart rate variability (HR/HRV) are associated with stress reaction, (ii) Higher basal sympathetic tone prior to stressful events is associated with higher performance, and (iii) Elite performers tend to exhibit greater increase in parasympathetic tone after a stressful event.

**Methods:** The current study assesses the predictive utility of post-stressful event HR/HRV measures, an under-studied time point in HR/HRV research, in the context of military personnel selection. Specifically, we examined the relationship between a comprehensive set of HR/HRV measures and established questionnaires related to stress tolerance, experimental evaluation of executive function during stress induction, and ecologically valid selection assessment data from a week-long Special Operations Forces selection course (N = 30).

**Results:** We found that post-stressful event HR/HRV measures generally had strong correlations with the neuroticism facet of the NEO personality inventory as well as the general and distress facets of the defensive reactivity questionnaire. HR/HRV measures correlated reliably with a change in executive function measured as a decrease in verbal fluency with exposure to a well-validated stressor. Finally, we observed a divergent pattern of correlation among elite and non-elite SOF candidates. Specifically, among elite candidates, parasympathetic nervous system (PNS) measures correlated positively and sympathetic nervous system (SNS) measures correlated negatively with evaluation of stress tolerance by experts and peers. This pattern was not present in non-elite candidates.

**Discussion:** Our findings demonstrate that post-stressful event HR/HRV data provide an objective non-invasive method to measure the recovery and arousal state in direct reaction to the stressful event and can be used as metrics of stress tolerance that could enhance selection of elite individuals and teams.

## 1 Introduction

Elite performance is characterized by extraordinary physiological capabilities accompanied by precise motor control (Williams and Reilly 2000; Ericsson et al., 2018). However, the physical skills necessary to perform a craft aren't sufficient to make a performer elite. One common factor among elite performers across fields is the ability to perform when confronted by strong emotion such as fear (Jones, Swain, and Hardy 1993; Hatfield and Hillman 2001; Bois et al., 2009). This ability to excel under stressful circumstances becomes increasingly important as the performer is recognized as an expert in their respective domain and the consequences of each performance become higher. Thus, accurately measuring stress tolerance could have wide-reaching implications for selection of elite performers in a variety of fields, such as better characterizing the biological determinants of elite performers (nature vs. nurture) and developing targeted training.

While the tests that measure various physical abilities are well-established, research and validation of cognitive factors pertinent to elite performance is still growing (Hardy, Jones, and Gould 2018; Scharfen and Memmert 2019). Under the conceptualization of *hot cognition* (Abelson 1963; Brand 1985), as opposed to “cold” cognition that deals with cognitive functioning in normal circumstances (Abelson 1963; Brand 1985), one’s ability to perform well with strong emotion can be seen as a product of *defensive reactivity* and *emotion regulation* (National Research Council, 2015). Defensive reactivity refers to one’s tendency to emotionally and physiologically react to fearful stimuli or stress, and it is believed to be a reflection of the amygdala-mediated defensive motivational system (Fanselow 1994; LeDoux 1995). Emotion regulation refers to one’s ability to “influence the occurrence, intensity, duration, and expression of emotion” (Campbell-Sills and Barlow 2007), such as resisting a temptation or trying not to show fear. In the influential process model of emotion regulation, first an emotionally relevant situation gives rise to one’s emotional state (i.e., defensive reactivity), and then one notices and appraises the emotion (i.e., emotion regulation) which results in the response (i.e., performance under stress) (Gross 2014).

Several self-report questionnaires that measure these and related constructs have been well-validated (e.g., Defensive Reactivity: (Kramer et al., 2012); Emotion Regulation Questionnaire: (Gross and John 2003; John and Gross 2004); Response Inventory: (Koh et al., 2001); Stress Tolerance Inventory: (Bland et al., 2012); Mental Toughness Questionnaire 48: (Clough, Earle, and Sewell 2002). However, because “desirable answers” can be deduced and given by the applicant, self-report measures have an inherent flaw in the context of selection assessment. Thus, developing and validating objective measures of stress response that are grounded in physiology and neuroscience that reliably predicts real-life performance under stress is an important research agenda (National Research Council, 2015).

Heart rate and heart rate variability (HR/HRV) are biomarkers which can be used to objectively evaluate changes in PNS and SNS activity of the autonomic nervous system (ANS) (Shaffer and Ginsberg 2017). HR/HRV can be gathered non-invasively (Castaldo et al., 2015; H-G. Kim et al., 2018) and have been used to detect response to both physical and mental stressors (Hamer and Steptoe 2007; Rosenberg et al., 2017; Roeser et al., 2012; H.-G. Kim et al., 2018). HRV has also been shown to be associated with the neural structures that are involved in the appraisal of threat and safety (Thayer, 2012). Thus, HRV can provide valuable information about an individual’s health, ANS function, and ability to respond to stress. The current study examines the utility of HR/HRV measures in the context of elite military personnel selection.

Given the link between HR/HRV and stress reaction, several studies in operational contexts have looked at HR/HRV measures as indicators of stress reaction and as predictors of performance in stressful events (Morgan et al., 2002; Jouanin et al., 2004; Morgan et al., 2007; Stanfill 2012; Tornero-Aguilera et al., 2017; J. F. Tornero-Aguilera, Robles-Pérez, and Clemente-Suárez 2018). A majority of these studies measured HR/HRV prior to a stressful event. For example, Morgan et al. showed that an individual’s perceived level of ‘burnout’ is significantly associated with increased parasympathetic tone, and top performers in a subsequent mentally and physically stressful task had higher pre-stressful event sympathetic tone (Morgan et al., 2002). Another study from the same research group reported that active-duty military personnel (men) enrolled in high intensity military training (Survival School in Experiments 1, 3 and Combat Diver Qualification Course in Experiment 2) showed a significant relationship between pre-stressful event low vagal tone [measured as High Frequency spectral power (.15–.40 Hz) and/or respiratory sinus arrhythmia] and superior performance. These findings suggest that vagal suppression prior to a high stress event is associated with enhanced performance, and enhanced performance may be related to emotion regulation and cognitive functioning (Morgan et al., 2007). Notably, in the context of military personnel selection, Stanfill (Stanfill 2012) showed significant correlations between Hell Week (a defining event during NAVY SEAL’s BUD/S training) completion and the standard deviation of all R-R intervals (SDNN). Contrary to expectation, SDNN had a positive correlation (r = .23) with completion of Hell Week.

**FIGURE 1:**
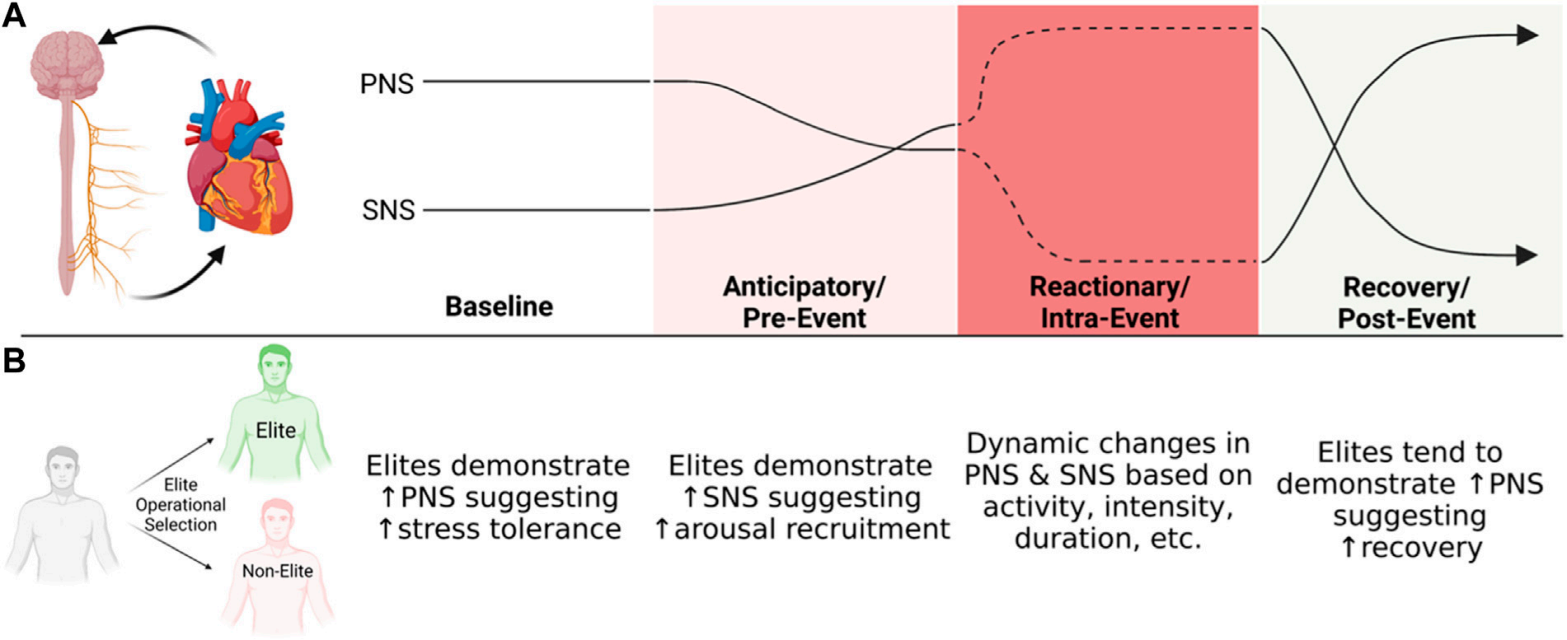
Schematic illustrations of how parasympathetic (PNS) and sympathetic (SNS) nervous system change throughout a temporal course of going through a stressful event **(A)** along with general characteristics of elite performers at each time point **(B)**.

Some studies included both pre- and post-stressful event HR/HRV measures. Jouanin et al. found that parasympathetic activity increased after rigorous physical and mental stress paradigms during ranger training (Jouanin et al., 2004). In another study comparing post-combat reaction among elite and non-elite operators, a significant increase in the low-frequency (LF) domain and a significant decrease in the high-frequency (HF) domain were found among elite operators during high physical and mental stress (J. F. Tornero-Aguilera, Robles-Pérez, and Clemente-Suárez 2018). Combined, these data suggest that i) HRV is associated with stress reaction, ii) higher basal sympathetic tone prior to stressful events is associated with higher performance, and iii) elite operators tend to exhibit greater increase in parasympathetic tone after a stressful event (see Figure 1 for schematic illustration of this general pattern).

Accordingly, using post-stressful event HR/HRV measures as indicators of stress reaction presents a unique opportunity to measure one’s response to an acute stress. However, very few studies have looked at the relationship between the post-stressful event HR/HRV measures and validated indices of stress reaction. Additionally, most studies only look at a limited number of time and/or frequency domain biomarkers, limiting the ability to understand autonomic responsiveness (Jouanin et al., 2004; Stanfill 2012); (Jouanin et al., 2004; Morgan et al., 2002; Morgan et al., 2007; Stanfill 2012; J F. Tornero-Aguilera, Robles-Pérez, and Clemente-Suárez 2018)). To address this gap, we examined the association between a comprehensive set of HR/HRV measures (20 features; see Table 1), and validated measures of stress response in elite level military performers during a week-long US SOF selection course. We hypothesized that greater post-stressful event parasympathetic tone would be associated with higher tendencies to react to stressors (as measured by established questionnaires), better ability to maintain executive function under stress, and superior stress tolerance. Given that sympathetic tone generally is negatively correlated with parasympathetic tone, we expected to observe the reverse patterns between the HR/HRV measures that reflect sympathetic and parasympathetic tones (i.e., SNS and PNS measures; see Section 3.1).

**TABLE 1:** HR/HRV parameters.

Parameter	Units	Description
Time Domain		
Mean RR	ms	The mean of RR interval, a metric of parasympathetic/sympathetic ratio
SDNN	Ms	Standard deviation of RR intervals, indicator of Vagal/Parasympathetic tone
Mean HR	bpm	Average heart rate, related to general fitness
Min & Max HR	bpm	Minimum and maximum heart rate, computed using N beat moving average (default value: N = 5)
Min-Max HR	bpm	Heart rate range
RMSSD	ms	Square root of the mean squared differences between successive RR intervals
NN50	#	Number of successive RR interval pairs that differ more than 50 m
pNN50	(%)	NN50 divided by the total number of RR intervals
Frequency Domain		
LF/HF	(no units)	Ratio of low frequency (LF: .04–.15 Hz) to high frequency (HF: .15–0.4 Hz) band powers
Total power	ms^2^	Absolute total power, sum of VLF, LF, and HF power
logLF, logHF, & logVLF	(no units)	Natural log transformed powers of low frequency (LF: 0.04–0.15 Hz), high frequency (HF: .15–.4 Hz), and very low frequency (VLF: .00–.04 Hz) bands
Non-linear		
SD1	ms	In Poincaré plot, the standard deviation perpendicular to the line-of-identity
SD2	ms	In Poincaré plot, the standard deviation along the line-of-identity
SD1/SD2	(no units)	Ratio of SD1 to SD2
DFA, α1	(no units)	In detrended fluctuation analysis, short term fluctuation slope
DFA, α2	(no units)	In detrended fluctuation analysis, long term fluctuation slope
Hybrid/Other		
Stress Index		Square root of Baevsky’s stress index
Tarvainen, Niskanen, and Lipponen (2014)		

## 2 Materials and methods

### 2.1 Participants and study design

In the current study, HR/HRV data were collected in conjunction with a week-long US military SOF selection course. Three types of data were used to assess the predictive utility of the post-selection-week HR/HRV measures: questionnaires, experimental evaluation of stress tolerance, and selection assessment data of stress tolerance. Resting-state electrocardiogram (ECG) data, from which we derived HR/HRV measures, were collected at the end of the physically and mentally challenging selection week. Thirty-eight SOF selection course candidates were included in the current study. Eight participants were excluded due to insufficient ECG data. The remaining thirty males were included in analyses (M_age_ = 24.5 years old, M_height_ = 69.2 inch/175.8 cm, M_weight_ = 179.7 lb/81.5 kg, M_BodyFatPercentage_ = 14.9%). Eleven were selected to serve in the SOF unit, and nineteen weren't selected.

Our research team collected physiological and phenotypic data prior to, during, and following four consecutive week long SOF selection courses over a year-long period. Our assessments included leadership and teamwork assessment using a novel individual and team shooting task in a virtual reality shooting simulator. While the analyses pertaining to these measures are out of the scope of the current paper, we included analysis of stress response on individual and team shooting performance out of the full set of data *a priori* (see Section 2.2 for the measures used in the current study), and examined their relationship with the post-selection week HR/HRV measures. We accessed assessment data from the SOF selection course for analysis. The list of all data and the HR/HRV measures which were included in the current study is reported in the following section. Approval of the oversight military Institutional Review Board (IRB) was obtained prior to starting the study. Informed consent was obtained from all study participants. The IRB protocol was followed without exception during performance of this research.

### 2.2 Data

#### 2.2.1 Heart rate and heart rate variability features

ECG data acquisition and HRV feature extraction. The day after the selection week was over, electrocardiographic (ECG) and electroencephalographic (EEG) data were acquired at the sampling rate of 300 Hz using a Wearable Sensing DSI-24 system. A pair of ECG electrodes were placed on the upper-back of each participant at the level of the heart, and about 2 inches right and left of the spine. The data collection was done in an approximately 1250ft2 room in a barrack that was converted into a testing room equipped with a shooting simulator (the results of the shoot sim performance is not reported in this paper). We ensured that the room stayed quiet while the ECG and EEG data were collected. Participants were seated during the recording with their eyes closed and the room’s light off. Participants were instructed to relax, stay awake, and refrain from moving during a 3 mins data collection. Caffeine consumption of any kind (e.g., coffee, energy drinks, pills, etc.) was not allowed. All participants followed the same protocol, and the ECG data were collected between 8 and 11 am)\. Raw data were filtered at 1.0–50.0 Hz. The resting-state ECG data were processed using Kubios HRV Premium, version 3.5.0 software (Tarvainen, Niskanen, and Lipponen 2014), to extract HR/HRV features as previously described (Koutnik et al., 2020). The Kubios trend removal method of smoothness priors regularization, automatic artifact correction, and automatic beat correction were applied. After the automatic pre-processing, all ECG signals were visually inspected and corrected for noise, software errors, and artifacts (e.g., skipped beats). Identified noise and artifacts were removed prior to analysis. In addition, following the emerging standard in the field, we have computed log transformed LF, HF, and VLF.

Variables used to assess heart rate variability are described below in Table 1.

#### 2.2.2 Well-validated questionnaires relevant to stress response

*NEO Neuroticism*. NEO personality inventory (PI) is the most widely used personality testing (McCrae et al., 2005). This personality trait has been associated with experiencing fear and anxiety at higher frequency and intensity (Thompson 2008) and has previously been used as a measure of stress tolerance (Steffens et al., 2013). We took the sum of the scores of NEO-PI’s neuroticism factor as our neuroticism measure (NEO N).

##### 2.2.2.1 Defensive reactivity questionnaire

DRQ is a hybrid questionnaire composed of questions from several well-established questionnaires (Emotionality-Activity-Sociability (EAS) Temperament Survey: (Buss and Robert, 2014); Fear Survey Schedule (FSS-III): (Arrindell, Emmelkamp, and January 1984); Psychopathic Personality Inventory: (Benning et al., 2003); Sensation Seeking Scale: (Zuckerman 1994); Tridimensional Personality Questionnaire: (Cloninger 1987)) that have the highest correlation with the startle response to sudden noise while viewing aversive pictures, one of the most established measures of one’s reactivity to stress (i.e., aversive startle potentiation: (Vaidyanathan, Patrick, and Bernat 2009). As a result of psychometric analysis involving exploratory and confirmatory factor analysis, three factors (sociability, distress, and stimulation seeking) making up one general factor on fear/fearlessness have been identified (Kramer et al., 2012). The items that were listed in a paper by Kramer et al. as the most representative of each subfactor (six items each) and the general factor (12 items) were used to compute the scores for each factor (DRQ General, DRQ Sociability, DRQ Distress, DRQ StimSeeking).

##### 2.2.2.2 Emotion regulation questionnaire

ERQ is a well-validated questionnaire measuring one’s tendency to regulate their emotion through generally adaptive *cognitive reappraisal* and generally maladaptive *expressive suppression* (Gross and John 2003; John and Gross 2004). The 10-item questionnaire gives two scores corresponding to one’s tendency to engage in reappraisal and suppression respectively (ERQ Reappraisal & ERQ Suppression).

#### 2.2.3 Experimental evaluation of stress tolerance

##### 2.2.3.1 Stress-induced change in verbal fluency

Verbal fluency task is an established measure of executive function (Shao et al., 2014). In the current study, participants completed four categories before and two categories after a stress induction. The pre/post-stress difference score of the normalized (z-score) average was computed as the index of stress-induced change in executive function, and higher scores indicate greater stress tolerance (Bhatia, Miyatsu, and Pirolli 2021). We used the Maastricht Acute Stress Test (MAST) as the stress induction. MAST is a clinically certified stress induction method that combines physical, cognitive, and social stressors, and reliably elicits glucocorticoid stress response as measured by salivary alpha-amylase and cortisol. Specifically, it involves immersing one’s hand under ice water and performing challenging cognitive tasks (e.g., mental arithmetic) under social evaluation and negative feedback (Smeets et al., 2012).

#### 2.2.4 Selection assessment data

##### 2.2.4.1 Physical fitness test-run

Candidates performance during the running portion of physical testing. A 1.5 or 3 mile run time (depending on the cohort) was converted to a scale of 1–5. Cardiovascular fitness has been previously linked to HRV and emotion regulation (Alderman and Olson 2014).

##### 2.2.4.2 Expert evaluation-ST

Senior officers and enlisted personnel’s’ subjective evaluation of candidates’ stress tolerance (ST) based upon observation during the selection course and rated on a scale of 1–5 (5 indicates greater tolerance).

##### 2.2.4.3 Peer perception-ST

Peer candidates’ subjective evaluation of candidates’ stress tolerance (ST) based upon peer evaluations at the end of the selection course and rated on a scale of 1-5.

### 2.3 Statistical approach

We examined the predictive utility of HR/HRV measures by comparing these variables to stress tolerance assessments that were collected before, during, and after the selection week (i.e., questionnaires, experimental evaluation of stress tolerance, and selection assessment data as described above). First, we used the R package corrplot to generate a correlation matrix of all HR/HRV measures. We used hclust (http://search.r-project.org/R/library/stats/html/hclust.html) with default parameter values to determine if data hierarchically clustered (Murtagh and Legendre 2014) into PNS and SNS groups. Second, we generated two correlation matrices, one for PNS HR/HRV and another for SNS HR/HRV measures to assess Pearson’s product-moment correlation with stress tolerance variables. These questionnaire, experimental evaluation of stress tolerance, and selection assessment results are described in the following sections. Finally, we explored the difference in HR/HRV measures among elite and non-elite participants by considering the selection assessment data separately based on the selection status (whether a given participant was selected to serve as an officer at the completion of the assessment week).

## 3 Results

### 3.1 Correlation among HR/HRV measures

**FIGURE 2:**
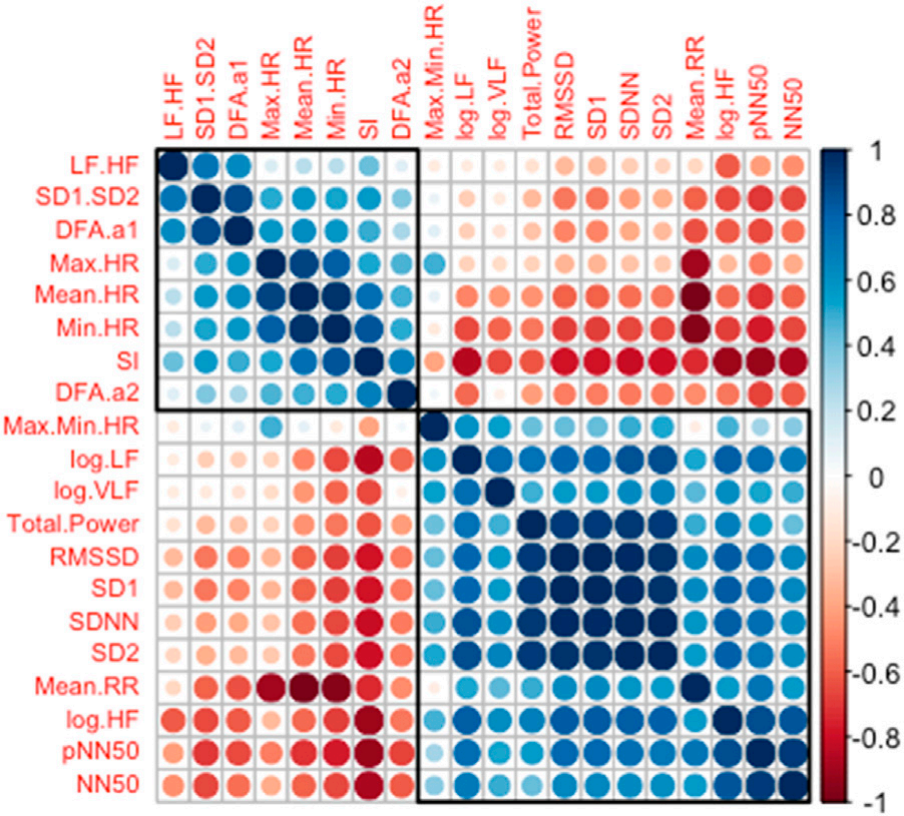
Hierarchically clustered correlation matrix among the HR/HRV measures. The color and the size of the circles indicate the strength of correlation with blue shades indicating positive and red shades indicating negative correlations. The black squares on top-left and bottom-right show the grouping based on the similarity and dissimilarity of each variable roughly clustering into the PNS (bottom-right) and the SNS (top-left) groups. Abbreviations: LF.HF, Ratio of low frequency (LF) to high frequency (HF) band powers; SD1.SD2, Ratio of Poincaré Perpendicular Standard Deviation (SD1) to Poincaré Parallel Standard Deviation (SD2); DFAα1, short-term detrended fluctuation analysis; HR, Heart Rate; SI, Stress Index; DFAα2, long-term detrended fluctuation analysis; Max.Min HR, Heart Rate Range; VLF, Very-Low Frequency; RMSSD, Root Mean Square of Successive Differences; SDNN, Standard Deviation between R-R intervals; RR, R to R interval in ECG rhythm; pNN50, percentage of successive R-R intervals that deviate greater than 50 ms; NN50, number of R-R intervals that deviate greater than 50 ms.

Figure 2 shows the hierarchically clustered and color-coded correlation matrix of the 20 composite HR/HRV measures. As can be seen from the strong correlation between the variables that are clustered on top-left and bottom right, these results demonstrate clear SNS (top-left) and PNS (bottom-right) clusters across HR/HRV across time, frequency, and non-linear HR/HRV measures. These group clusters are consistent with previous findings (Tarvainen, Niskanen, and Lipponen 2014; Shaffer and Ginsberg 2017; Minarini 2020; Pham et al., 2021). Of note, while logVLF, DFA, and SD measures aren't generally specific to SNS or PNS, we clustered these measures based on their PNS/SNS grouping relationship demonstrated in Figure 2 for all subsequent analyses. These data demonstrate intra-PNS/SNS measurement convergent validity utilizing unbiased cluster analyses of all 20 composite biomarkers in a SOF selection course. We acknowledge that there is a discussion on whether HRV directly measures SNS activities (Shaffer and Ginsberg 2017) or they are measures of parasympathetic withdrawal (Shaffer and Ginsberg 2017; Hayano and Yuda 2019). However, we elected to refer to the cluster variables as SNS variables for ease of understanding. The sample size, Pearson r, and the *p*-value corresponding to the correlations among the HR/HRV measures are reported in the Supplementary Tables S1–S3.

### 3.2 Correlation between the PNS measures and questionnaires

Table 2 shows the correlation between the PNS HR/HRV measures and all non-HR/HRV variables included in the current study (i.e., questionnaires, stress-induced change in verbal fluency, and selection assessment data). Regarding the questionnaire variables, there was a reliable negative relationship between the PNS measures and the neuroticism facet of the NEO personality inventory. All twelve PNS variables showed a negative correlation with an average of *r* =−.39, and seven of them were significant (Max-Min HR: *r*(26) =−.42, *p* = .03; Total Power: r(26) = −.40, *p* = .04; RMSSD: *r*(26) =−.45, *p* = .02; SD1: *r*(26) = −.45, *p* = .02; SDNN: *r*(26) = −.41, *p* = .03; pNN50: *r*(26) = −.48, *p* = .01; NN50: *r*(26) = −.40, *p* = .04). There was no significant correlation between the PNS measures and the emotion regulation questionnaire with average correlations of *r* = −.22 with the reappraisal facet and *r* = −.07 with the suppression facet. While there were no significant correlation between the PNS measures and the sociability and stimulation seeking facets of the defensive reactivity questionnaire (average *r* = −.14 for sociability and .06 for stimulation seeking), there was a negative relationship between the PNS measures and the general facet as well as between the PNS measures and the distress facet. For the general facet, the average correlation was *r* = −.27 with four of them showing significant or marginally significant relationship (Max-Min HR: *r*(27) = −.40, *p* = .03; Total power: *r*(27) = −.37, *p* = .05; SDNN: *r*(27) = −.32, *p* = .09; SD2: *r*(27) = −.33, *p* = .08). For the distress facet the average correlation was *r* = −.48 with eleven of them showing significant relationship (Max-Min HR: *r*(27) = −.60, *p* < .001; logLF: r(27) = −.58, *p* < .001; logVLF: r(27) = −.49, *p* = .007; Total power: r(27) = −.52, *p* = .004; RMSSD: *r*(27) = −.52, *p* = .004; SD1: *r*(27) = −.52, *p* = .004; SDNN: *r*(27) = −.59, *p* = .002; SD2: *r*(27) = −.57, *p* = .001; logHF: r(27) = -.42, *p* = .02; qNN50: *r*(27) = -.40, *p* = .04; NN50: *r*(27) = −.37, *p* = .05). These data suggest that higher parasympathetic activity following a multi-day physically and mentally stressful event is associated with a lower sensitivity to stress as measured through established questionnaires.

**TABLE 2:** Correlations between the PNS HR/HRV measures and trait/performance measures.

Pearson’s correlations													
Variable		Max-min HR	log LF	Log VLF	Total power	RMSSD	SD1	SDNN	SD2	Mean RR	Log HF	pNN50	NN50
VF	N	20	20	20	20	20	20	20	20	20	20	20	20
	Pearson’s r	−.02	.414	.129	.383	.344	.344	.359	.359	.52*	.253	.227	.142
	*p*-value	.933	.07	.588	.096	.138	.138	.12	.12	.019	.281	.335	.55
NEO N	N	28	28	28	28	28	28	28	28	28	28	28	28
	Pearson’s r	−.416*	−.366	−.26	−.395*	−.453*	−.453*	−.406*	−.366	−.259	−.43*	−.481*	−.397*
	*p*-value	.028	.056	.182	.037	.016	.016	.032	.055	.184	.023	.01	.036
ERQ Reappraisal	N	26	26	26	26	26	26	26	26	26	26	26	26
	Pearson’s r	−.251	−.377	−.046	–.235	−.231	−.231	−.272	−.288	.092	−.24	−.282	−.248
	*p*-value	.217	.058	.824	.247	.256	.256	.178	.153	.656	.237	.164	.222
ERQ Suppression	N	26	26	26	26	26	26	26	26	26	26	26	26
	Pearson’s r	−.196	−.113	.039	−.011	−.047	−.047	−.046	−.043	.025	−.188	−.133	−.077
	*p*-value	.337	.583	.85	.956	.819	.818	.824	.835	.905	.359	.517	.708
DRQ General	N	29	29	29	29	29	29	29	29	29	29	29	29
	Pearson’s r	−.4*	−.307	−.144	−.372*	−.302	−.302	−.324	−.33	−.067	−.215	−.243	−.275
	*p*-value	.031	.106	.455	.047	.112	.112	.086	.08	.73	.263	.204	.148
DRQ Sociability	N	29	29	29	29	29	29	29	29	29	29	29	29
	Pearson’s r	−.266	−.225	−.121	−.175	−.123	−.123	−.175	−.195	.043	−.068	−.097	−.122
	*p*-value	.163	.241	.531	.365	.525	.525	.363	.312	.823	.727	.616	.527
DRQ Distress	N	29	29	29	29	29	29	29	29	29	29	29	29
	Pearson’s r	−.597***	−.584***	−.49**	−.519**	−.517**	−.517**	−.558**	−.571**	−.25	−.421	−.389*	−.369*
	*p*-value	< .001	< .001	.007	.004	.004	.004	.002	.001	.19	.023	.037	.049
DRQ StimSeeking	N	29	29	29	29	29	29	29	29	29	29	29	29
	Pearson’s r	.128	.07	−.027	.035	.096	.096	.094	.091	−.111	.173	.07	.047
	*p*-value	.509	.72	.889	.855	.622	.622	.629	.637	.568	.369	.719	.81
Physical Fitness Test-Run	N	28	28	28	28	28	28	28	28	28	28	28	28
	Pearson’s r	.021	.179	.215	.017	−.098	−.097	.016	.069	.06	−.142	-.089	–.098
	*p*-value	.916	.363	.272	.93	.622	.622	.936	.728	.762	.47	.651	.619
Expert Evaluation-ST	N	28	28	28	28	28	28	28	28	28	28	28	28
	Pearson’s r	−.054	.136	.216	.13	.098	.097	.168	.202	.068	.061	7.724e –5	−.041
	*p*-value	.785	.491	.269	.51	.621	.622	.393	.303	.733	.76	1	.835
Peer Perception-ST	N	29	29	29	29	29	29	29	29	29	29	29	29
	Pearson’s r	−.287	–.112	−.018	–.025	.039	.039	.035	.037	.096	.073	.044	−.017
	*p*-value	.131	.563	.927	.899	.839	.84	.858	.848	.619	.708	.821	.931

* *p* < .05, ** *p* < .01, *** *p* < .001

VF, stress-induced change in verbal fluency; NEO N, NEO neuroticism; ERQ, emotion regulation questionnaire; DRQ, defensive reactivity questionnaire; ST, stress tolerance; Max-Min HR, heart rate range; LF, low frequency; VLF, Very-Low Frequency; RMSSD, root mean square of successive differences; SD1, Poincaré Perpendicular Standard Deviation; SDNN, Standard Deviation between R-R intervals; SD2, Poincaré Parallel Standard Deviation; RR, R to R interval in ECG, rhythm; HF, high frequency; pNN50, percentage of successive R-R intervals that deviate greater than 50 ms; NN50, number of R-R intervals that deviate greater than 50 ms.

### 3.3 Correlation between the SNS measures and questionnaires

Table 3 shows the correlation between the SNS HR/HRV measures and all non-HR/HRV variables included in the current study. None of the SNS measures significantly correlated with the neuroticism facet of the NEO personality inventory. DFA a2 was the only measure that significantly correlated with the reappraisal facet of the ERQ, *r*(24) = .45, *p* = .02, and none of the SNS measures significantly correlated with the suppression facet of the ERQ. Regarding the DR questionnaire, two SNS measures significantly or marginally significantly correlated with the distress facet (Minimum HR: r(27) = .36, *p* = .06; Stress Index: *r*(27) = .47, *p* = .01), LF/HF power marginally significantly correlated with the stimulation seeking facet, *r*(27) = −.37, *p* = .05, and none of the SNS measures significantly correlated with the general or the sociability facet. These data suggest that higher sympathetic activity following a multi-day physically and mentally stressful event is associated with a greater sensitivity to stress as measured through established questionnaires although this association is not as strong as the association between the parasympathetic activity and the questionnaire measures (3.2).

**TABLE 3:** Correlations between the SNS HR/HRV measures and trait/performance measures.

Pearson’s correlations									
Variable		LF/HF	SD1/SD2	DFA a1	Max HR	Mean HR	Min HR	SI	DFA a2
VF	N	20	20	20	20	20	20	20	20
	Pearson’s r	.138	−.042	−.074	−.475*	−.51*	−.542*	−.406	−.43
	*p*-value	.561	.861	.755	.034	.021	.014	.076	.058
NEO N	N	28	28	28	28	28	28	28	28
	Pearson’s r	.153	.242	.29	.027	.209	.307	.347	.152
	*p*-value	.438	.214	.134	.892	.285	.112	.07	.44
ERQ Reappraisal	N	26	26	26	26	26	26	26	26
	Pearson’s r	.029	−.064	−.092	−.163	−.109	−.022	.243	.446*
	*p*-value	.89	.754	.655	.427	.597	.916	.232	.022
ERQ Suppression	N	26	26	26	26	26	26	26	26
	Pearson’s r	.293	.158	.181	−.116	−.062	−.004	.076	.15
	*p*-value	.147	.44	.377	.573	.763	.984	.713	.463
DRQ General	N	29	29	29	29	29	29	29	29
	Pearson’s r	−.015	.066	−.068	−.103	.081	.146	.258	.247
	*p*-value	.937	.734	.728	.595	.677	.449	.176	.197
DRQ Sociability	N	29	29	29	29	29	29	29	29
	Pearson’s r	−.131	−.224	−.266	−.109	−.032	.051	.129	.267
	*p*-value	.499	.242	.164	.573	.868	.793	.505	.161
DRQ Distress	N	29	29	29	29	29	29	29	29
	Pearson’s r	−.106	.038	−.026	−.03	.247	.359	.472*	.205
	*p*-value	.584	.845	.893	.877	.197	.056	.01	.287
DRQ StimSeeking	N	29	29	29	29	29	29	29	29
	Pearson’s r	−.366	−.084	.007	.139	.105	.075	−.135	−.211
	*p*-value	.051	.665	.972	.471	.588	.7	.484	.271
Physical Fitness Test-Run	N	28	28	28	28	28	28	28	28
	Pearson’s r	.329	.373	.378*	−.089	−.073	−.117	−.043	−.211
	*p*-value	.088	.051	.047	.652	.713	.555	.827	.281
Expert Evaluation-ST	N	28	28	28	28	28	28	28	28
	Pearson’s r	.054	.145	.155	−.091	−.067	−.073	−.17	−.13
	*p*-value	.785	.461	.432	.645	.733	.711	.386	.509
Peer Perception-ST	N	29	29	29	29	29	29	29	29
	Pearson’s r	−.239	−.216	−.303	−.197	−.093	−.049	−.069	.002
	*p*-value	.212	.261	.11	.307	.632	.801	.721	.991

* *p* < .05, ** *p* < .01, *** *p* < .001.

VF, stress-induced change in verbal fluency; NEO N, NEO neuroticism; ERQ, emotion regulation questionnaire; DRQ, defensive reactivity questionnaire; ST, stress tolerance; LF, low frequency; HF, high frequency; SD1, Poincaré Perpendicular Standard Deviation; SD2, Poincaré Parallel Standard Deviation; DFAα1, short-term detrended fluctuation analysis; HR, heart rate; SI, stress index; DFAα2, long-term detrended fluctuation analysis.

### 3.4 Correlation between the PNS/SNS measures and stress-induced change in VF

There was a positive relationship between PNS measures and stress tolerance in executive function. Eleven out of the twelve PNS measures showed a positive correlation with an average of *r* = .29, and three of them were significant or marginally significant despite the low sample size (logLF: r(18) = .41, *p* = .07; Total power: r(18) = .38, *p* = .10; Mean RR: *r*(18) = .52, *p* = .02). SNS measures generally had a negative correlation with stress tolerance in executive function with an average of *r* = −.29 and five of them showing significant or marginally significant correlation despite the small sample size (Max HR: *r*(18) = −.48, *p* = .03; Mean HR: *r*(18) =−.51, *p* = .02; Minimum HR: *r*(18) = −.54, *p* = .01; Stress Index: *r*(18) = −−.41, *p* = .08; DFA a2: *r*(18) = −.43, *p* = .06). These data show that higher parasympathetic activity following a multi-day physically and mentally stressful event is associated with a greater ability to maintain executive function under acute stress whereas higher sympathetic activity following a stressful event is associated with a lesser ability to maintain executive function under acute stress.

### 3.5 Correlation between the PNS/SNS measures and selection assessment data

None of the PNS measures significantly correlated with Physical Fitness Test-Run while three SNS measures significantly or marginally significantly correlated with it (LF/HF: r(26) = .33, *p* = .09; SD1/SD2: r(26) = .37, *p* = .05; DFA a1: *r*(26) = .38, *p* = .05). These data replicated prior findings associating cardiovascular fitness with HRV and stress response (Hamer and Steptoe 2007).

**FIGURE 3:**
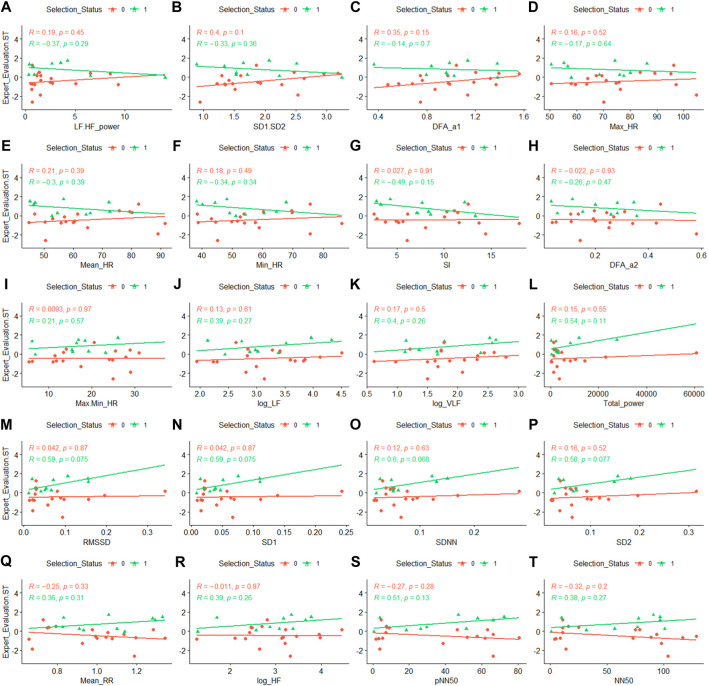
Correlation between Expert Evaluation-ST and HRV metrics by Selection Status. Selection Status of 0, shown in red with circular data points, indicates a non-selected participant, and Selection Status of 1, shown in green with triangular data points, indicates a selected participant. Abbreviations: ST, stress tolerance; HRV, Heart Rate Variability. Panel descriptions **(A)** LF.HF, Ratio of low frequency (LF) to high frequency (HF) band powers; **(B)** SD1.SD2, Ratio of Poincaré Perpendicular Standard Deviation (SD1) to Poincaré Parallel Standard Deviation (SD2); **(C)** DFAα1, short-term detrended fluctuation analysis; **(D)** Max Heart Rate; **(E)** Mean Heart Rate; **(F)** Minimum Heart Rate; **(G)** SI, Stress Index; **(H)** DFAα2, long-term detrended fluctuation analysis; **(I)** Max.Min HR, Heart Rate Range; **(J)** logLF, natural log of LF; **(K)** logVLF, natural log of Very-Low Frequency; **(L)** Total Power; **(M)** RMSSD, Root Mean Square of Successive Differences; **(N)** SD1; **(O)** SDNN, Standard Deviation between R-R intervals; **(P)** SD2; **(Q)** Mean RR, R to R interval in ECG rhythm; **(R)** logHF, natural log of HF; **(S)** pNN50, percentage of successive R-R intervals that deviate greater than 50 ms; **(T)** NN50, number of R-R intervals that deviate greater than 50 ms.

**FIGURE 4:**
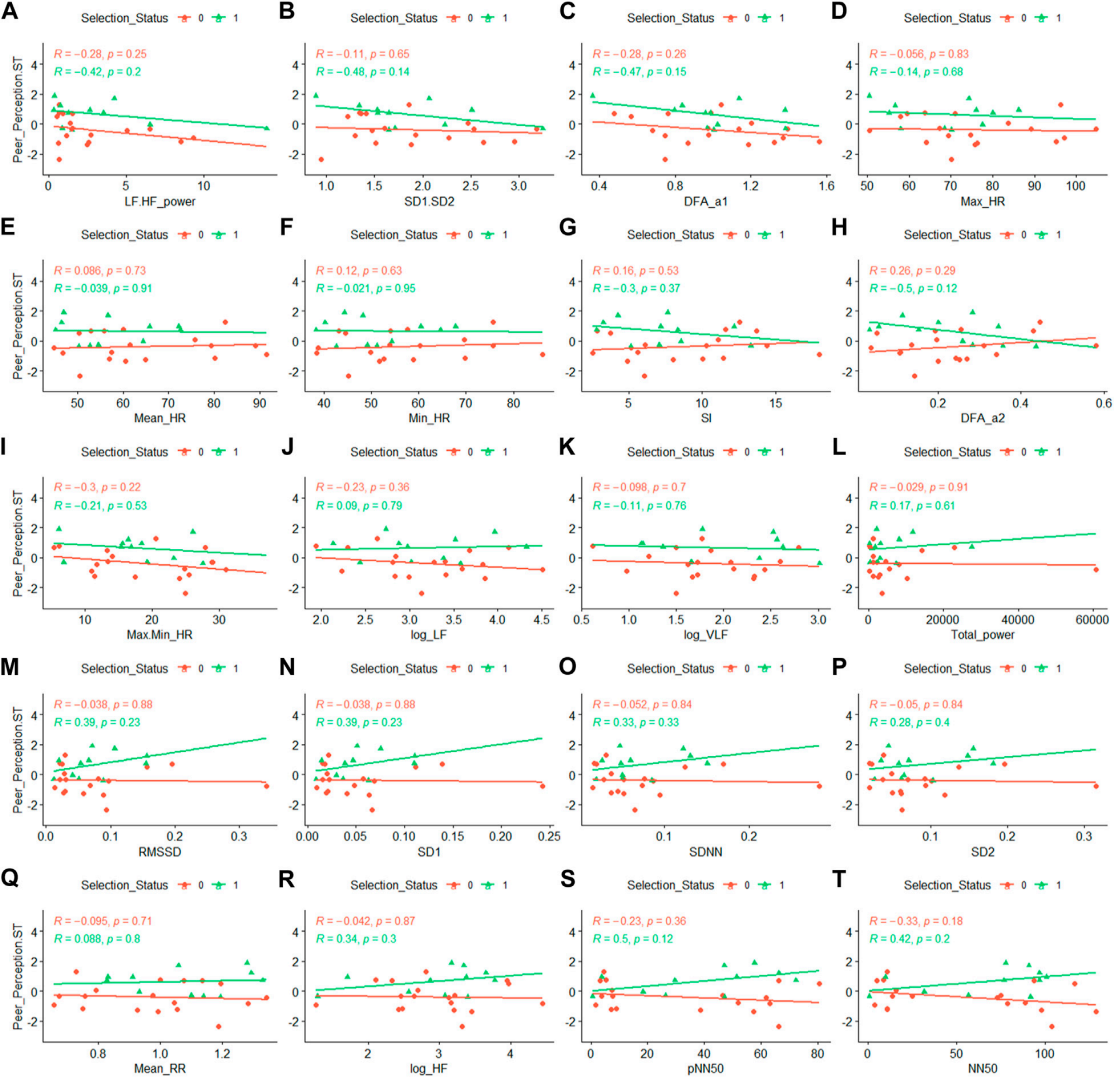
Correlation between Peer Perception-ST and HRV metrics by Selection Status. Selection Status of 0, shown in red with circular data points, indicates a non-selected participant, and Selection Status of 1, shown in green with triangular data points, indicates a selected participant. Abbreviations: ST, stress tolerance; HRV, Heart Rate Variability. Panel descriptions **(A)** LF.HF, Ratio of low frequency (LF) to high frequency (HF) band powers; **(B)** SD1.SD2, Ratio of Poincaré Perpendicular Standard Deviation (SD1) to Poincaré Parallel Standard Deviation (SD2); **(C)** DFAα1, short-term detrended fluctuation analysis; **(D)** Max Heart Rate; **(E)** Mean Heart Rate; **(F)** Minimum Heart Rate; **(G)** SI, Stress Index; **(H)** DFAα2, long-term detrended fluctuation analysis; **(I)** Max.Min HR, Heart Rate Range; **(J)** logLF, natural log of LF; **(K)** logVLF, natural log of Very-Low Frequency; **(L)** Total Power; **(M)** RMSSD, Root Mean Square of Successive Differences; **(N)** SD1; **(O)** SDNN, Standard Deviation between R-R intervals; **(P)** SD2; **(Q)** Mean RR, R to R interval in ECG rhythm; **(R)** logHF, natural log of HF; **(S)** pNN50, percentage of successive R-R intervals that deviate greater than 50 ms; **(T)** NN50, number of R-R intervals that deviate greater than 50 ms.

None of the individual correlation between the Expert Evaluation-ST and the HR/HRV measures nor Peer Perception-ST and the HR/HRV measures reached significance. To explore the relationship between the HR/HRV measures and the stress tolerance observed during selection week, we broke down these correlations by whether a given participant was selected (yellow lines in Figures 3, 4) or not selected (blue lines) to serve in the SOF unit at the end of selection week. Interestingly, for both Expert Evaluation-ST and Peer Perception-ST, a consistent pattern emerged. Among those selected, the PNS measures (twelve plots labeled i to t) correlated positively with Expert Evaluation-ST (average *r* = .37) and Peer Perception-ST (average *r* = .22), and the SNS measures (thirteen plots labeled a to h) correlated negatively with Expert Evaluation-ST (average *r* = −.27) and Peer Perception-ST (average *r* = −.30). Among those not selected, this pattern did not emerge (PNS: average *r* = −.002 for Expert Evaluation-ST & average *r* = −.13 for Peer Perception-ST; SNS: average *r* = .19 for Expert Evaluation-ST & average *r* = −.01 for Peer Perception-ST). This demonstrates that post-stressful event PNS activity in selection candidates is associated with elite performance and stress tolerance. This also suggests a biological difference in how elite performers autonomically respond to stressful stimuli.

## 4 Discussion

### 4.1 Summary of findings

In the current study, we assessed the predictive utility of post-stressful event HR/HRV measures in the context of a SOF selection course. Specifically, we examined the relationship between a comprehensive set of HR/HRV measures with established questionnaires related to stress tolerance, experimental evaluation of stress tolerance in cognitive function, and ecologically valid selection assessment data.

We found a reliable negative relationship between the neuroticism facet of the NEO personality inventory and the PNS measures.This finding suggests that people with lower neuroticism experience greater parasympathetic activation after a stressful event potentially because of a greater ability to recover from a greater perceived stress from the event. Previously, our team demonstrated that individuals who are “psychologically less fit” do not recover (parasympathetic rebound) as effectively after stressful events (A. P. Koutnik et al., 2014), suggesting that elite military personnel may have elevated psychological fitness which contributes to parasympathetic rebound after stress. This finding is an important addition to the literature characterizing the dynamic nature of elites autonomic states(Ode et al., 2010). While elite performers exhibit greater SNS activation prior to a stressful event, suggesting their ability to recruit arousal (Morgan et al., 2002), our findings show that they also show elevated PNS activation after a stressful event. In general, there was no correlation between the HR/HRV measures and the ERQ (except DFA a2-ERQ-suppression). The lack of a relationship may indicate that the tendency to employ particular types of cognitive emotion regulation strategies (reappraisal and suppression) is more relevant to the immediate experience of stress during the stressful event, but less so for post-stressful event autonomic states.

While there were no significant correlations between the HR/HRV measures and the sociability and stimulation seeking facets of the DR questionnaire, there was a negative relationship between the PNS measures and the general and distress facets. In addition, a small number of SNS measures positively correlated with the distress facet and negatively correlated with the stimulation seeking facet. These results are consistent with the previous meta-analytic findings associating HRV with the neural structures that are involved in the appraisal of threat and safety (Thayer, 2012) as the defensive reactivity questionnaires measure the function of the amygdala-mediated defensive system. These results also align with our prior research that demonstrated individuals with higher tendency to experience distress are also likely to experience dysautonomia in response to a stressful event (Koutnik et al., 2014).

Critically, both the PNS and SNS measures significantly predicted stress tolerance in executive functioning which was assessed by the well-established verbal fluency task before and after a clinically certified stress induction that combined physical, cognitive, and social stressors (Smeets et al., 2012). This finding demonstrates the utility of the HR/HRV measures in predicting acute change in cognition due to stress.

We did not observe a significant relationship between the HR/HRV measures and the experts’ and peers’ perception of a candidate’s stress tolerance during selection week. It is important to note that these measures are subjective in nature and likely incorporate broader perception of a given candidate (e.g., how likable/professional/effortful he was). Indeed, the correlation between the different qualities (e.g., stress tolerance, professionalism, demonstrated effort, teamwork) from the same selection assessment expert evaluation data were generally high (*r* > .6). Moreover, these measures of stress tolerance included observation of physical performance under stress, in contrast to the stress-induced change in executive function (VF-ST) which is a pure cognitive performance measure. This difference could help explain why correlations between HR/HRV measures and VF-ST were higher than the correlations between HR/HRV and selection assessment stress tolerance data. Interestingly, there was a divergent pattern of correlation among the selected and non-selected in our sample. Specifically, among the elites (i.e., participants who were selected), PNS measures correlated positively and the SNS measures correlated negatively with expert and peer evaluation of stress tolerance. This pattern did not emerge among non-elite candidates (i.e., those who weren't selected). Although these findings should be considered with caution due to a small sample size (selected: N = 11; non-selected: N = 19), the pattern of response within elite versus non-elite personnel across multiple metrics of stress, suggests that elite personnel might be biologically different from non-elites in how they respond to stress. This is further supported by prior literature suggesting potential inherent biological differences contributing to performance differences (D. S. Kim, Wheeler, and Ashley 2022).

### 4.2 Pre-/post- stressful-event HR/HRV measures and HR/HRV dynamics

As described in the introduction, most of the past research investigating the predictive utilities of HR/HRV measures collected HR/HRV data before a stressful event and showed mixed results in terms of their predictive utility. Specifically, one study associated low vagal tone (i.e., low HF power) with superior performance (Morgan et al., 2007) while another associated high vagal tone (i.e., high SDNN) with superior performance (Stanfill 2012). It is possible that this discrepancy reflects the difference between the measures used to infer autonomic state (i.e., HF power vs. SDNN), but given the high correlation between these two HRV measures observed in the current study and elsewhere (Otzenberger et al., 1998) this isn't likely. It is possible that this ambiguity is related to the timing of HR/HRV data collection. In Morgan et al.’s study, the HR/HRV data were collected just a few days before the stressful event at the site where the stressful event took place. In Stanfill’s study, the HR/HRV measures were collected 2 months before the stressful event to avoid “anticipatory anxiety.” Given ANS’s sensitivity to one’s mental state and growing evidence supporting the dynamic nature of HRV (Ode et al., 2010), researchers carefully consider the timing and the setting in which HR/HRV data are collected.

Our findings demonstrate that HR/HRV data post-stressful events provide an objective non-invasive method to measure the recovery and arousal state in direct reaction to the stressful event. However, the post-stressful event HR/HRV measures also have inherent issues. For example, post-stressful event HR/HRV data often cannot be collected from candidates who did not complete the event. Although we did not collect pre-event HR/HRV data due to logistical constraints, in future analysis, we plan to integrate continuous monitoring of HR/HRV measures to better understand the ANS preparation and response dynamics in relation to the event. Future research examining the relationship between HR/HRV measures and performance during a mentally and physically demanding event could benefit from a similar approach.

### 4.3 Post stressful-event resting-state HR/HRV as stress tolerance assessment tool

The current study expanded upon previous research, and demonstrated the promise of post-stressful event HR/HRV measures as a tool to assess stress tolerance. The post-stressful event HR/HRV measures generally had strong correlation with the neuroticism facet of NEO personality inventory as well as the general and distress facets of the defensive reactivity questionnaire. In terms of psychometrics, HR/HRV measures had good concurrent validity with the established instruments measuring stress tolerance. HR/HRV measures also correlated reliably with a stringent test of cognitive resilience under stress, the experimental evaluation of executive function measured using the well-validated verbal fluency task administered before and after a well-characterized stress induction. Critically, from an operational standpoint, HR/HRV measures can be inexpensively and passively collected using wearable devices without the need for lengthy questionnaires or complicated experimental procedures. As such, additional research exploring the utility of post-stressful-event HR/HRV is warranted.

### 4.4 Limitations

The main limitations of the study are the small sample size, skewed gender distribution, and the short (3 min) ECG data collection window. Given the small sample size stemming from the highly applied and specialized nature of our study, we weren't able to adjust for multiple comparisons in our correlational analyses. Thus, it is possible that some of our positive findings include Type 1 error (i.e., false positives). However, the general observation that relatively clear SNS and PNS grouping emerged, the variables within the grouping showed similar trend, and the variables across the grouping showed opposite trend tempers the concerns that there are many Type 1 errors in our analyses. All our participants were male. As such, whether the findings would generalize to female elite performers is uncertain. In the domain of elite athletes, it has been shown that male and female athletes differ in their HRV (Berkoff et al., 2007) and stress response (Ong 2017; Patócs et al., 2016). Future studies would benefit from considering gender as a variable of interest in examining HRV and stress response among elite military personnel. Finally, logistical limitations stemming from the highly applied and specialized nature of our study necessitated a short (3 min) ECG recording window. While a longer ECG recording (e.g., 5 min) would have been ideal, extracting reliable HRV measures from shorter recording is possible as evidenced in a respected review (Shaffer and Ginsberg 2017) as well as our team’s previous work (Koutnik et al., 2014; Koutnik et al., 2020).

## Data Availability

The anonymized raw data supporting the conclusion of this article will be made available by the authors upon formal request, without undue reservation.
