# Supplementary material for: Resting-state heart rate variability after stressful events as a measure of stress tolerance among elite performers
Source: Front Physiol. 2023 Jan 4;13:1070285. doi: 10.3389/fphys.2022.1070285 (PMC9848589; doi:10.3389/fphys.2022.1070285)
Supplement: Supplementary file 1 [file Table1.docx]

| **Supplemental Table 1**  *Correlations among the PNS HR/HRV measures (n=30)* | | | | | | | | | | | | | | | | | | | | | | | | |
| --- | --- | --- | --- | --- | --- | --- | --- | --- | --- | --- | --- | --- | --- | --- | --- | --- | --- | --- | --- | --- | --- | --- | --- | --- |
| Pearson's Correlations | | | | | | | | | | | | | | | | | | | | | | | | |
| **Variable** |  | **Max-Min HR** | | **log LF** | | **log VLF** | | **Total Power** | | **RMSSD** | | **SD1** | | **SDNN** | | **SD2** | | **Mean RR** | | **log HF** | | **pNN50** | | **NN50** |
| Max-Min HR | Pearson's r | — |  |  |  |  |  |  |  |  |  |  |  |  |  |  |  |  |  |  |  |  |  |  |
|  | p-value | — |  |  |  |  |  |  |  |  |  |  |  |  |  |  |  |  |  |  |  |  |  |  |
| log LF | Pearson's r | 0.591 | *** | — |  |  |  |  |  |  |  |  |  |  |  |  |  |  |  |  |  |  |  |  |
|  | p-value | < .001 |  | — |  |  |  |  |  |  |  |  |  |  |  |  |  |  |  |  |  |  |  |  |
| log VLF | Pearson's r | 0.546 | ** | 0.759 | *** | — |  |  |  |  |  |  |  |  |  |  |  |  |  |  |  |  |  |  |
|  | p-value | 0.002 |  | < .001 |  | — |  |  |  |  |  |  |  |  |  |  |  |  |  |  |  |  |  |  |
| Total Power | Pearson's r | 0.414 | * | 0.735 | *** | 0.482 | ** | — |  |  |  |  |  |  |  |  |  |  |  |  |  |  |  |  |
|  | p-value | 0.023 |  | < .001 |  | 0.007 |  | — |  |  |  |  |  |  |  |  |  |  |  |  |  |  |  |  |
| RMSSD | Pearson's r | 0.428 | * | 0.79 | *** | 0.561 | ** | 0.93 | *** | — |  |  |  |  |  |  |  |  |  |  |  |  |  |  |
|  | p-value | 0.018 |  | < .001 |  | 0.001 |  | < .001 |  | — |  |  |  |  |  |  |  |  |  |  |  |  |  |  |
| SD1 | Pearson's r | 0.428 | * | 0.789 | *** | 0.561 | ** | 0.931 | *** | 1 | *** | — |  |  |  |  |  |  |  |  |  |  |  |  |
|  | p-value | 0.018 |  | < .001 |  | 0.001 |  | < .001 |  | < .001 |  | — |  |  |  |  |  |  |  |  |  |  |  |  |
| SDNN | Pearson's r | 0.492 | ** | 0.856 | *** | 0.625 | *** | 0.943 | *** | 0.984 | *** | 0.984 | *** | — |  |  |  |  |  |  |  |  |  |  |
|  | p-value | 0.006 |  | < .001 |  | < .001 |  | < .001 |  | < .001 |  | < .001 |  | — |  |  |  |  |  |  |  |  |  |  |
| SD2 | Pearson's r | 0.515 | ** | 0.879 | *** | 0.65 | *** | 0.937 | *** | 0.964 | *** | 0.964 | *** | 0.996 | *** | — |  |  |  |  |  |  |  |  |
|  | p-value | 0.004 |  | < .001 |  | < .001 |  | < .001 |  | < .001 |  | < .001 |  | < .001 |  | — |  |  |  |  |  |  |  |  |
| Mean RR | Pearson's r | -0.091 |  | 0.511 | ** | 0.441 | * | 0.495 | ** | 0.628 | *** | 0.628 | *** | 0.585 | *** | 0.556 | ** | — |  |  |  |  |  |  |
|  | p-value | 0.632 |  | 0.004 |  | 0.015 |  | 0.005 |  | < .001 |  | < .001 |  | < .001 |  | 0.001 |  | — |  |  |  |  |  |  |
| log HF | Pearson's r | 0.473 | ** | 0.819 | *** | 0.615 | *** | 0.672 | *** | 0.828 | *** | 0.828 | *** | 0.819 | *** | 0.806 | *** | 0.569 | ** | — |  |  |  |  |
|  | p-value | 0.008 |  | < .001 |  | < .001 |  | < .001 |  | < .001 |  | < .001 |  | < .001 |  | < .001 |  | 0.001 |  | — |  |  |  |  |
| pNN50 | Pearson's r | 0.292 |  | 0.757 | *** | 0.527 | ** | 0.558 | ** | 0.772 | *** | 0.772 | *** | 0.742 | *** | 0.718 | *** | 0.722 | *** | 0.874 | *** | — |  |  |
|  | p-value | 0.117 |  | < .001 |  | 0.003 |  | 0.001 |  | < .001 |  | < .001 |  | < .001 |  | < .001 |  | < .001 |  | < .001 |  | — |  |  |
| NN50 | Pearson's r | 0.352 |  | 0.696 | *** | 0.493 | ** | 0.422 | * | 0.641 | *** | 0.64 | *** | 0.626 | *** | 0.614 | *** | 0.561 | ** | 0.842 | *** | 0.938 | *** | — |
|  | p-value | 0.056 |  | < .001 |  | 0.006 |  | 0.02 |  | < .001 |  | < .001 |  | < .001 |  | < .001 |  | 0.001 |  | < .001 |  | < .001 |  | — |
| * p < .05, ** p < .01, *** p < .001 | | | | | | | | | | | | | | | | | | | | | | | | |

*Notes.* Abbreviations: Max-Min HR, Heart Rate Range; LF, Low Frequency; VLF, Very-Low Frequency; RMSSD, Root Mean Square of Successive Differences; SD1, Poincaré Perpendicular Standard Deviation; SDNN, Standard Deviation between R-R intervals; SD2, Poincaré Parallel Standard Deviation; RR, R to R interval in ECG rhythm; HF, High Frequency; pNN50, percentage of successive R-R intervals that deviate greater than 50ms; NN50, number of R-R intervals that deviate greater than 50ms.

**Supplemental Table 2**

*Correlations among the SNS HR/HRV measures (n=30)*

| Pearson's Correlations | | | | | | | | | | | | | | | | | |
| --- | --- | --- | --- | --- | --- | --- | --- | --- | --- | --- | --- | --- | --- | --- | --- | --- | --- |
| **Variable** |  | **LF/HF** | | **SD1/SD2** | | **DFA a1** | | **Max HR** | | **Mean HR** | | **Min HR** | | **SI** | | **DFA a2** | |
| LF/HF | Pearson's r | — |  |  |  |  |  |  |  |  |  |  |  |  |  |  |  |
|  | p-value | — |  |  |  |  |  |  |  |  |  |  |  |  |  |  |  |
| SD1/SD2 | Pearson's r | 0.722 | *** | — |  |  |  |  |  |  |  |  |  |  |  |  |  |
|  | p-value | < .001 |  | — |  |  |  |  |  |  |  |  |  |  |  |  |  |
| DFA a1 | Pearson's r | 0.624 | *** | 0.889 | *** | — |  |  |  |  |  |  |  |  |  |  |  |
|  | p-value | < .001 |  | < .001 |  | — |  |  |  |  |  |  |  |  |  |  |  |
| Max HR | Pearson's r | 0.14 |  | 0.502 | ** | 0.572 | *** | — |  |  |  |  |  |  |  |  |  |
|  | p-value | 0.462 |  | 0.005 |  | < .001 |  | — |  |  |  |  |  |  |  |  |  |
| Mean HR | Pearson's r | 0.238 |  | 0.577 | *** | 0.613 | *** | 0.904 | *** | — |  |  |  |  |  |  |  |
|  | p-value | 0.205 |  | < .001 |  | < .001 |  | < .001 |  | — |  |  |  |  |  |  |  |
| Min HR | Pearson's r | 0.23 |  | 0.521 | ** | 0.574 | *** | 0.813 | *** | 0.965 | *** | — |  |  |  |  |  |
|  | p-value | 0.221 |  | 0.003 |  | < .001 |  | < .001 |  | < .001 |  | — |  |  |  |  |  |
| SI | Pearson's r | 0.412 | * | 0.563 | ** | 0.498 | ** | 0.513 | ** | 0.747 | *** | 0.842 | *** | — |  |  |  |
|  | p-value | 0.024 |  | 0.001 |  | 0.005 |  | 0.004 |  | < .001 |  | < .001 |  | — |  |  |  |
| DFA a2 | Pearson's r | 0.119 |  | 0.372 | * | 0.273 |  | 0.467 | ** | 0.488 | ** | 0.503 | ** | 0.676 | *** | — |  |
|  | p-value | 0.53 |  | 0.043 |  | 0.144 |  | 0.009 |  | 0.006 |  | 0.005 |  | < .001 |  | — |  |
| * p < .05, ** p < .01, *** p < .001 | | | | | | | | | | | | | | | | | |

*Notes.* Abbreviations: LF, Low Frequency; HF, High Frequency; SD1, Poincaré Perpendicular Standard Deviation; SD2, Poincaré Parallel Standard Deviation; DFAα1, short-term detrended fluctuation analysis; HR, Heart Rate; SI, Stress Index; DFAα2, long-term detrended fluctuation analysis.

**Supplemental Table 3**

*Correlations between the PNS and SNS HR/HRV measures (n=30)*

| Pearson's Correlations | | | | | | | | | | | | | | | | | |
| --- | --- | --- | --- | --- | --- | --- | --- | --- | --- | --- | --- | --- | --- | --- | --- | --- | --- |
| **Variable** |  | **LF/HF** | | **SD1/SD2** | | **DFA a1** | | **Max HR** | | **Mean HR** | | **Min HR** | | **SI** | | **DFA a2** | |
| Max-Min HR | Pearson's r | -0.107 |  | 0.075 |  | 0.114 |  | 0.485 | ** | 0.093 |  | -0.114 |  | -0.391 | * | 0.042 |  |
|  | p-value | 0.572 |  | 0.695 |  | 0.549 |  | 0.007 |  | 0.623 |  | 0.548 |  | 0.033 |  | 0.826 |  |
| log LF | Pearson's r | -0.108 |  | -0.247 |  | -0.231 |  | -0.225 |  | -0.485 | ** | -0.649 | *** | -0.849 | *** | -0.568 | ** |
|  | p-value | 0.572 |  | 0.188 |  | 0.22 |  | 0.233 |  | 0.007 |  | < .001 |  | < .001 |  | 0.001 |  |
| log VLF | Pearson's r | -0.105 |  | -0.12 |  | -0.148 |  | -0.184 |  | -0.436 | * | -0.572 | *** | -0.648 | *** | -0.08 |  |
|  | p-value | 0.581 |  | 0.529 |  | 0.436 |  | 0.33 |  | 0.016 |  | < .001 |  | < .001 |  | 0.676 |  |
| Total Power | Pearson's r | -0.16 |  | -0.32 |  | -0.287 |  | -0.222 |  | -0.446 | * | -0.527 | ** | -0.614 | *** | -0.419 | * |
|  | p-value | 0.398 |  | 0.085 |  | 0.124 |  | 0.239 |  | 0.013 |  | 0.003 |  | < .001 |  | 0.021 |  |
| RMSSD | Pearson's r | -0.318 |  | -0.525 | ** | -0.485 | ** | -0.339 |  | -0.587 | *** | -0.67 | *** | -0.788 | *** | -0.502 | ** |
|  | p-value | 0.087 |  | 0.003 |  | 0.007 |  | 0.067 |  | < .001 |  | < .001 |  | < .001 |  | 0.005 |  |
| SD1 | Pearson's r | -0.318 |  | -0.525 | ** | -0.485 | ** | -0.339 |  | -0.587 | *** | -0.67 | *** | -0.788 | *** | -0.502 | ** |
|  | p-value | 0.087 |  | 0.003 |  | 0.007 |  | 0.067 |  | < .001 |  | < .001 |  | < .001 |  | 0.005 |  |
| SDNN | Pearson's r | -0.248 |  | -0.414 | * | -0.371 | * | -0.289 |  | -0.55 | ** | -0.656 | *** | -0.801 | *** | -0.517 | ** |
|  | p-value | 0.187 |  | 0.023 |  | 0.043 |  | 0.121 |  | 0.002 |  | < .001 |  | < .001 |  | 0.003 |  |
| SD2 | Pearson's r | -0.213 |  | -0.357 |  | -0.312 |  | -0.262 |  | -0.524 | ** | -0.64 | *** | -0.799 | *** | -0.518 | ** |
|  | p-value | 0.258 |  | 0.053 |  | 0.093 |  | 0.162 |  | 0.003 |  | < .001 |  | < .001 |  | 0.003 |  |
| Mean RR | Pearson's r | -0.202 |  | -0.58 | *** | -0.621 | *** | -0.89 | *** | -0.98 | *** | -0.951 | *** | -0.725 | *** | -0.465 | * |
|  | p-value | 0.284 |  | < .001 |  | < .001 |  | < .001 |  | < .001 |  | < .001 |  | < .001 |  | 0.01 |  |
| log HF | Pearson's r | -0.608 | *** | -0.648 | *** | -0.609 | *** | -0.314 |  | -0.566 | ** | -0.672 | *** | -0.904 | *** | -0.529 | ** |
|  | p-value | < .001 |  | < .001 |  | < .001 |  | 0.091 |  | 0.001 |  | < .001 |  | < .001 |  | 0.003 |  |
| pNN50 | Pearson's r | -0.412 | * | -0.7 | *** | -0.642 | *** | -0.503 | ** | -0.704 | *** | -0.766 | *** | -0.913 | *** | -0.664 | *** |
|  | p-value | 0.024 |  | < .001 |  | < .001 |  | 0.005 |  | < .001 |  | < .001 |  | < .001 |  | < .001 |  |
| NN50 | Pearson's r | -0.456 | * | -0.65 | *** | -0.549 | ** | -0.368 | * | -0.582 | *** | -0.653 | *** | -0.877 | *** | -0.602 | *** |
|  | p-value | 0.011 |  | < .001 |  | 0.002 |  | 0.045 |  | < .001 |  | < .001 |  | < .001 |  | < .001 |  |
| * p < .05, ** p < .01, *** p < .001 | | | | | | | | | | | | | | | | | |

*Notes.* Abbreviations: Max-Min HR, Heart Rate Range; LF, Low Frequency; VLF, Very-Low Frequency; RMSSD, Root Mean Square of Successive Differences; SD1, Poincaré Perpendicular Standard Deviation; SDNN, Standard Deviation between R-R intervals; SD2, Poincaré Parallel Standard Deviation; RR, R to R interval in ECG rhythm; HF, High Frequency; pNN50, percentage of successive R-R intervals that deviate greater than 50ms; NN50, number of R-R intervals that deviate greater than 50ms; DFAα1, short-term detrended fluctuation analysis; SI, Stress Index; DFAα2, long-term detrended fluctuation
